# Dangers from Drugs Purchased from Peddlers

**Published:** 1914-06

**Authors:** 


**Dangers from Drugs purchased from Peddlers.** Edward H. Marsh, Medical Times, April 14, 1914, discusses the recent death of seven inmates of the Los Angeles County Hospital, from salvarsan injections and ascribes it to poor quality of the drug. He also discusses the general proposition of purchasing drugs from unreliable sources. The persons who sold the ''salvarsan'' have refused to state its source. It is a safe rule when life is at stake, not to be too economic but to deal with reliable firms. And, a fairly safe rule as to reliability, is to deal with such firms as do not peddle their publicity from house to house by circulars but who maintain space that can be consulted as needed, in medical journals. Journal advertising is, of course, not a positive guarantee of reliability but it elicits criticism which, in the long run determines the survival of the fit.

